# Molecular Diversity of the Casein Gene Cluster in Bovidae: Insights from SNP Microarray Analysis

**DOI:** 10.3390/ani14203034

**Published:** 2024-10-19

**Authors:** Tadeusz Malewski, Stanisław Kamiński, Jan Śmiełowski, Kamil Oleński, Wiesław Bogdanowicz

**Affiliations:** 1 Department of Molecular and Biometric Techniques, Museum and Institute of Zoology, Polish Academy of Sciences, 00-818 Warszawa, Poland; tmalewski@miiz.waw.pl; 2 Department of Animal Genetics, University of Warmia and Mazury, 10-718 Olsztyn, Poland; stachel@uwm.edu.pl (S.K.); kamel@uwm.edu.pl (K.O.); 3 Independent Researcher, 60-809 Poznań, Poland; jan.smielowski@gmail.com

**Keywords:** casein gene cluster, Bovidae, genotyping, evolution, SNP microarray

## Abstract

**Simple Summary:**

This study explores the genetic diversity of the casein gene cluster, a key component in milk protein production, across various Bovidae species, including cattle, buffaloes, and antelopes. The lack of genetic data for wild bovids hampers the understanding of milk protein expression and the improvement of dairy breeds. The researchers assessed the feasibility of using a cattle-specific SNP microarray to analyze genetic variation in 12 Bovidae taxa, representing 5 different subfamilies. The positive results enabled the examination of genetic variations and their potential impact on milk protein production. The findings revealed high genetic diversity in these genes among domestic cattle and certain wild species, such as the lechwe, suggesting variations in milk protein expression. The study demonstrated that cattle-derived SNP chips are effective tools for examining genetic diversity in other Bovidae species. Moreover, the relatively low cost of SNP microarrays and the ability of third-party companies to scan them make genomic analysis applicable to a wide range of research areas.

**Abstract:**

The casein gene cluster spans 250 to 350 kb across mammalian species and is flanked by non-coding DNA with largely unknown functions. These regions likely harbor elements regulating the expression of the 4 casein genes. In Bovidae, this cluster is well studied in domestic cattle and to a lesser extent in zebu and water buffalo. This study used a cattle-specific SNP microarray to analyze 12 Bovidae taxa and estimate casein gene cluster variability across 5 bovid subfamilies. Genotyping identified 126 SNPs covering the entire casein gene cluster and 2 Mb of upstream and downstream regions. Dairy cattle, watusi, and zebu showed the highest polymorphism: 63.7–68.2% in the 5′-upstream region, 35.6–40.0% in the casein cluster, and 40.4–89.4% in the 3′-downstream region. Among wild bovids, only a ‘semi-aquatic’ lechwe revealed high polymorphism similar to cattle. Other species exhibited lower variability, ranging from 9.1–27.3% in the 5′-upstream, 8.9–20.0% in the casein, and 4.2–10.6% in the 3′-downstream regions. For the first time, genome variability data were obtained for impala, waterbuck, and lechwe. It appears that higher variability in cattle’s casein gene cluster may relate to its intense expression. This study confirms the effectiveness of cattle-derived microarrays for genotyping Bovidae.

## 1. Introduction

Lactation is a key aspect of mammalian life history. In the family of cloven-hoofed mammals, Bovidae, caseins are the most abundant milk proteins. They provide nursing infants with essential amino acids, calcium, phosphorus, and potentially bioactive peptides, and their composition varies among species, driven by reproductive strategies, the developmental needs of the young, and the environment of the maternal–infant pair. Bovidae, which includes species with diverse milk yields and compositions—differing in its content of protein, fat, and solids [1]—rely on caseins for critical nutrition and development. Given their nutritional significance, Bovidae contribute to approximately 96% of the world’s milk production, primarily from cattle (81%), water buffaloes (15%), goats (2.3%), and sheep (1.4%) [2].

Caseins belong to the secretory calcium-binding phosphoproteins (SCPPs) family and are believed to have evolved from ancestral genes through duplication and exon changes. These ancestral genes derived from other *Scpp* genes, either the ameloblast-associated gene (*Odam*), the SCPP-Pro-Gln-rich 1 gene (*Scpppq1*), or the follicular dendritic cell secreted peptide gene (*Fdcsp*) [3].

The bovid casein gene cluster spans 250 kb and consists of 7 genes: α-S1-casein (*Csn1s1*), β-casein (*Csn2*), histatin (*Htn*), statherin (*Stath*), α-S2-casein (*Csn1s2*), the ameloblast-associated gene (*Odam*), and κ-casein (*Csn3*) [4,5]. This gene cluster is highly conserved in mammals, although some differences in its organization and content have been reported. For example, in humans, two histatin genes (*Htn1* and *Htn3*) and the follicular dendritic cell secreted peptide gene (*Fdcsp*) are located between the casein genes. In contrast, the gene clusters of mice and rats lack the statherin and histatin genes [6]. Among the casein gene cluster members, the *Odam* gene is proposed to be the ancestral gene from which all other casein genes evolved [3].

Among bovids, the exon–intron structure of 4 casein genes has been analyzed in cattle and, to some extent, in water buffaloes [7,8,9]. Additionally, the evolution of individual casein genes, such as β-casein [10] and κ-casein [11], has been studied. Interspecies comparisons have shown that κ-casein is the most conserved of the casein genes, likely due to its essential role in stabilizing casein micelles [12]. Casein genes are highly expressed in epithelial cells during late pregnancy, lactation, and early involution of the mammary gland [13,14,15]. These genes are separated and flanked by stretches of non-coding DNA with mostly unknown functions, believed to contain elements that regulate their spatio-temporal expression patterns [4,16,17,18]. Sequence characterization of the *Csn3* promoter region has revealed several variations in putative transcription factor binding sites that affect gene transcription in cattle [19], zebu, and water buffalo [20].

Although Bovidae comprises more than 140 species [21], detailed data on the casein gene cluster are limited to a few species, such as *Bos taurus* [4,12,22], *Bos indicus* [4,20,23]; *Bubalus bubalis* [7,20], *Capra hircus* [24], and *Ovis aries* [25], with most research focusing on milk composition (as, e.g., in the case of *Hippotragus niger* [26], *Damaliscus pygargus phillipsi*, *Connochaetes gnou*, *C. taurinus* [27], *Taurotragus oryx*, *Kobus leche* [28], *Aepyceros melampus*, and *Damaliscus lunatus lunatus* [29]) rather than on genetic structure. Broader studies on casein gene structures are limited by the high cost of next-generation sequencing (NGS). They can be extended by microarray technology, which is a more cost-effective solution. This study aims to (1) analyze the diversity of the casein gene cluster in both domestic and wild Bovidae species using a bovine SNP microarray and (2) identify the specific segments of the casein gene cluster wherein differences in SNP frequencies are most significant. Identifying these segments could offer new insights into the well-known variations in milk biosynthesis, particularly the low production in wild bovids and the high production in their domesticated counterparts.

## 2. Materials and Methods

**Figure 1:**
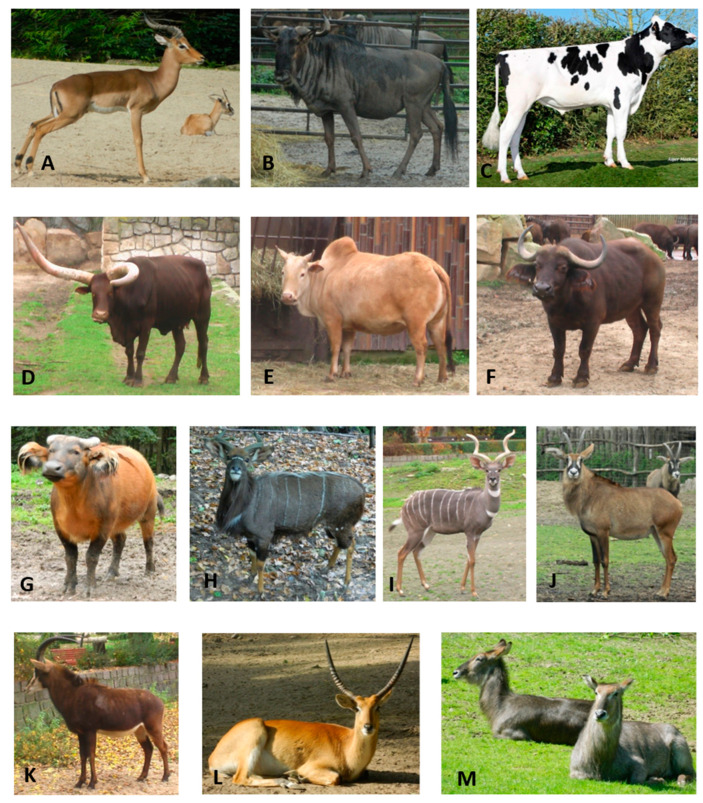
Bovidae taxa included in the analysis of casein gene cluster diversity: (**A**) impala (*Aepyceros melampus*), (**B**) blue wildebeest (*Connochaetes taurinus*), (**C**) domestic cattle (*Bos taurus taurus*, Holstein–Friesian breed), (**D**) watusi (*B. t. taurus*), (**E**) zebu (*Bos indicus*), (**F**) African buffalo (*Syncerus caffer caffer*), (**G**) African forest buffalo (*S. c. nanus*), (**H**) nyala (*Tragelaphus angassi*), (**I**) lesser kudu (*T. imbersis*), (**J**) roan antelope (*Hippotragus equinus*), (**K**) sable antelope (*H. niger*), (**L**) red lechwe (*Kobus leche*), and (**M**) waterbuck (*K. ellipsiprymnus*). Photographs: (**A**,**B**,**D**–**M**)—J. Śmiełowski; (**C**)—M. Marciniak.

Blood samples were collected from 76 animals representing 5 subfamilies of the family Bovidae: (i) Aepycerotinae: *Aepyceros melampus* (Lichtenstein, 1812) (*n* = 5); (ii) Alcelaphinae: *Connochaetes taurinus* (Burchell, 1823) (*n* = 5); (iii) Bovinae: *Bos taurus* Linnaeus, 1758 (Holstein breed, *n* = 12 and watusi, *n* = 5), *B. indicus* Linnaeus, 1758 (*n* = 5), *Syncerus caffer* (Sparrman, 1779) (*n* = 9), *Tragelaphus angasii* Angas, 1849 (*n* = 5), *T. imberbis* (Blyth, 1869) (*n* = 5); (iv) Hippotraginae (*n* = 5): *Hippotragus equinus* (É. Geoffroy Saint-Hilaire, 1803) (*n* = 5), *H. niger* (Harris, 1838) (*n* = 5); (v) Reduncinae: *Kobus leche* Gray, 1850 (*n* = 5), and *K. ellipsiprymnus* (Ogilby, 1833) (*n* = 5) (Figure 1). These animals came from the zoo in Dvůr Králové nad Labem, Czechia. Their ancestors originated from diverse regions in Africa. They were imported to the Dvůr Králové zoo, Czechia in the 1970s and bred to avoid inbreeding by exchanging reproductive individuals among zoological gardens, thus adhering to the guidelines of the European Association of Zoos and Aquaria (EAZA). Blood samples were taken during routine veterinary inspections in 2016–2020 and frozen in −20 °C until DNA preparation.

Genomic DNA was isolated by the use of a NucleoSpin Tissue Mini kit (Macherey-Nagel, Düren, Germany), according to the manufacturer’s protocol. All animals were genotyped using Illumina Bovine MDv2 Chip (Illumina, San Diego, CA, USA), according to the manufacturer’s protocol. The raw SNP data passed standard quality control in Illumina GenomeStudio software ver. 2011.1 [30], following the protocol provided by Illumina. The quality control process included checking for cluster separation, call frequency, AB T mean, rep errors (repeatability of genotype calls), het excess (excess calls of heterozygotes), minor frequency, and gender estimation. The P-C errors (Mendelian inheritance errors) option was not used because our populations did not consist of parents and offspring. Initial data cleanup removed poorly performing SNPs or/and samples giving an insufficient number of informative genotypes. Monomorphic SNPs, SNPs showing a significant deviation (*p* < 0.001) from the Hardy–Weinberg equilibrium (HWE), and SNPs with a minor allele frequency (MAF) < 0.01 were also removed from further analysis. Additionally, the similarity of 10 randomly selected Illumina probe sequences related to the casein gene cluster (Table 1) was checked using BLASTn against the available genome assembly of the analyzed species.

GenAlEx v. 6.501 was used to calculate genetic diversity indices, including observed (Ho) and expected (He) heterozygosities, expected unbiased heterozygosity (uHe), and the fixation index (Fst) [31,32]. The bovine UMD3.1 genome assembly was used to annotate the detected SNPs. Phylogenetic relationships were reconstructed using MEGA X ver. 10.0.5 [33].

## 3. Results

**Figure 2:**
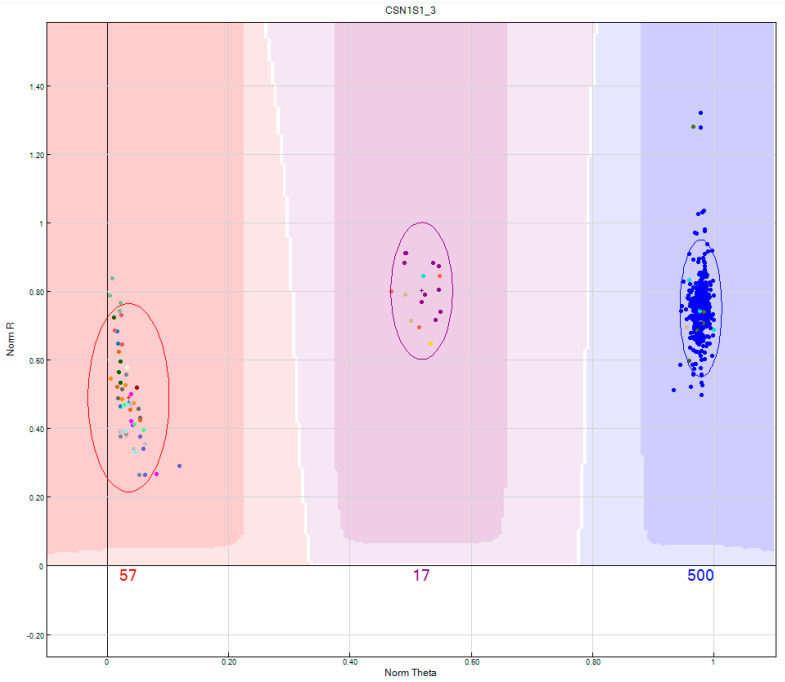
Example of the cluster quality of a single SNP in the Illumina Bovine EuroG_MDv2 Chip, located within the casein α-S1 gene (*Csn1s1*). Dots of different colors represent individuals from different Bovidae taxa. The clusters are clearly separated into three distinct areas, indicating that clustering is unambiguous and SNP genotypes were identified reliably. To enhance cluster quality, Bovidae species were analyzed alongside a large number of *B. taurus* (Holstein breed) individuals (blue dots).

Raw SNP data from the casein gene cluster and surrounding 5′- and 3′-regions, which passed the standard quality control in Illumina Genome Studio software, were inspected using scatter plots (Figure 2). Failed or suboptimal SNPs were excluded from further analyses. After editing, 45,556 SNPs were obtained, including 126 located in the casein gene cluster and within 2 Mb of their 5′-upstream and 3′-downstream regions.

### 3.1. SNP Call Rates

No-call SNPs can result from experimental errors, lack of probe sequence similarity to the DNA of the examined species, or incompatibility of the nucleotide at the 3′ end. To address this issue, the similarities between probe sequences and genomic sequences were examined (Table 2). Out of the 10 probe sequences analyzed across eight species, only one probe (BTA-111108-no-rs) showed no similarity in one of them (*A. melampus*). In all other cases, sequence identity ranged from 86% to 100%. For probes that were successfully called in all samples, sequence identity ranged from 88% to 100%; for those called in some samples, it ranged from 92% to 94%. In 4 out of 10 probes, the last nucleotide at the 3′ end was incompatible with the analyzed genomic sequence. An example of such a probe is presented in Table 3.

SNP genotyping of samples using the Bovine BeadChip revealed that all SNP call rates ranged from 83.0% to 100.0%. Similarly, the call rate for the casein gene cluster ranged from 82.9% to 100.0% (Table 4).

### 3.2. SNP Polymorphism

The percentage of SNPs polymorphism in the casein cluster ranged from 8.9% to 55.6%. High levels of polymorphism were detected in *K. leche* (55.6%), *B. taurus* (domestic cattle―37.8%, watusi―40.0%), and *B. indicus* (zebu―35.6%). In other species, the polymorphism was significantly lower; even in *S. caffer* belonging to the same subfamily, the polymorphism was ca. 2.5-fold lower (15.6%). The lowest level of polymorphism was detected in *A. melampus* (8.9%) (Table 5).

In the case of the 5′-upstream region, the percentage of polymorphism ranged from 9.1% in *K. ellipsiprymnus* and *T. imberbis* to 68.2% in domestic cattle and watusi. For the 3′-downstream region, this ranged from 4.3% in *K. ellipsiprymnus* to 89.4% in domestic cattle. The level of polymorphism in the regions flanking the casein cluster was generally similar to the polymorphism within the cluster itself in most species. However, in domestic cattle, watusi, and zebu, the polymorphism in the 5′-upstream region was 1.7- to 1.8-fold higher than within the casein cluster. Similarly, in these breeds, the 3′-flanking region was also more polymorphic than the casein cluster (by 2.4-, 1.3-, and 1.1-fold, respectively). In contrast, among wild Bovidae (except *A. melampus*), the polymorphism in the 3′-downstream region was 1.3- to 3.1-fold lower than in the casein cluster. Furthermore, in all species except domestic cattle, the polymorphism in the 3′-downstream region was 1.1- to 4.3-fold lower than in the 5′-upstream region. Notably, in domestic cattle, the 3′-downstream region was 1.3-fold more polymorphic than the 5′-upstream region (Table 5).

A total of 58 SNPs were identified in the casein gene cluster, with 45 in exons, 6 in introns, and 7 in intergenic regions (Table S1). The lowest level of polymorphism in exons (8.6%) was observed in *A. melampus*, while the highest was found in species of the genus *Bos* (domestic cattle—34.3%, watusi—37.1%) and in *K. leche* (57.1%). Within the genus *Kobus*, polymorphism varied significantly from 14.3% in *K. ellipsiprymnus* to 57.1% in *K. leche*. Generally, SNPs in introns were more conserved than those in exons across most species and varied from 0.0% (*A. melampus*) to 50.0% (domestic cattle and zebu). In domestic cattle, zebu, and water buffalo, intronic polymorphism was higher than exonic polymorphism. For *C. taurinus* and *T. angasii*, SNPs in intergenic sequences were more conserved compared to those in exons (Table 6).

### 3.3. Genetic Diversity Indices

Heterozygosity observed (Ho) in the 5′-upstream region ranged from 0.045 to 0.373. The lowest heterozygosity was detected in the genus *Tragelaphus* (0.045 for *T. imbersis* and 0.066 for *T. angasii*), while the highest value was found in the genus *Bos* (0.223, 0.232, and 0.373 for domestic cattle, zebu, and watusi, respectively). Similar patterns were noted in the casein gene cluster, with the lowest Ho values observed in *Tragelaphus* (0.042 and 0.093 for *T. imbersis* and *T. angasii*, respectively) and the highest in *Bos* (0.122, 0.151, and 0.188 for domestic cattle, zebu, and watusi, respectively). In the 3′-downstream region, Ho values were highest in the genus *Bos* (0.143, 0.235, and 0.350 for zebu, watusi, and domestic cattle, respectively), while the lowest values were not observed in the genus *Tragelaphus*, as might be expected (see above), but were observed in the representatives of three other genera, *K. ellipsiprymnus* (0.040), *H. equinus* (0.041), and *C. taurinus* (0.056). Total fixation index values were negative for all analyzed regions (−0.230, −0.307, and −0.202), indicating an excess of heterozygotes in most cases (Table 6).

### 3.4. Casein Gene Cluster and Flanking Regions-Based Phylogeny

**Figure 3:**
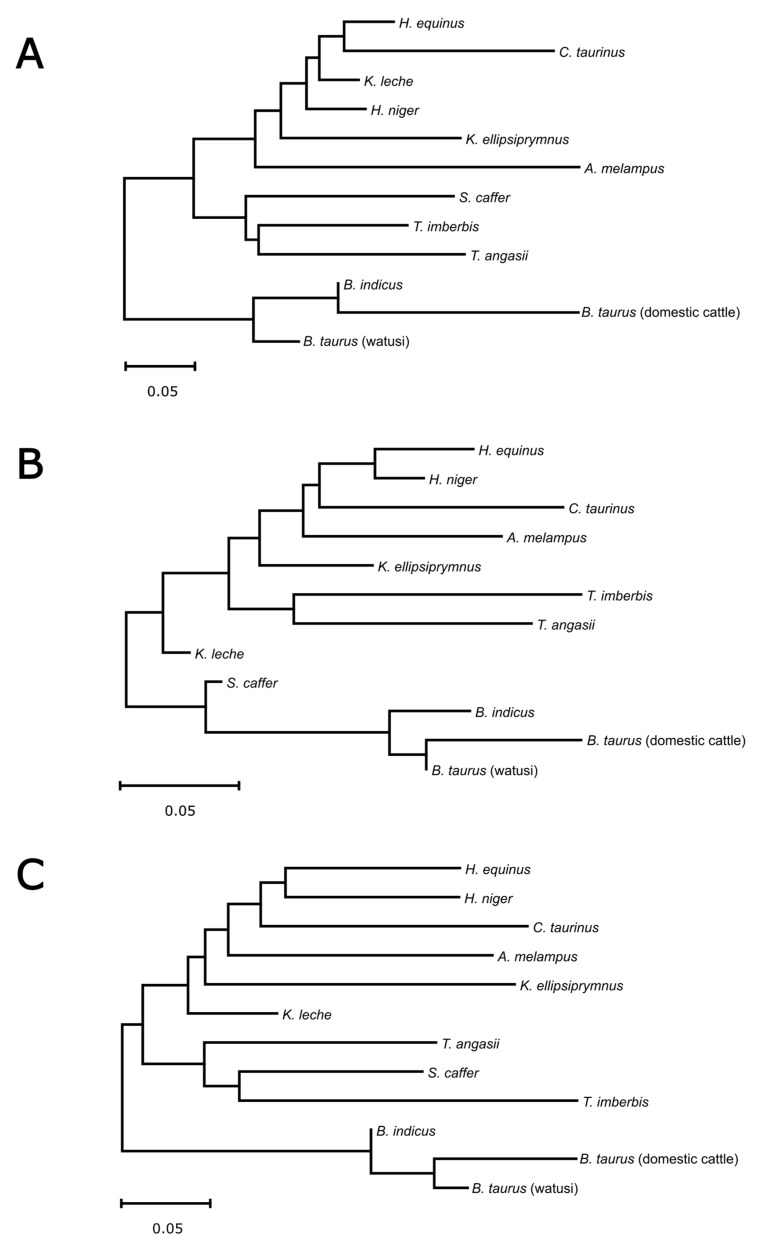
The dendrogram of the Bovidae species based on the Nei unbiased genetic distance inferred from SNPs located in the (**A**) 5′-upstream region, (**B**) casein gene cluster, and (**C**) 3′-downstream region.

Phylogenetic reconstruction based on SNPs located in the 5′-upstream region, casein genes cluster, and 3′-downstream region, using genetic distance matrix, yielded two main clusters (Figure 3). One cluster consisted of domestic cattle, watusi, and zebu (*Bos* cluster), while all remaining species were grouped in the second cluster. On the graph inferred from the casein gene cluster, *S. caffer* was positioned as a sister taxon to the other *Bos* taxa (Figure 3B; see also Table S2).

## 4. Discussion

The genetic polymorphisms of casein genes have been widely studied in domestic cattle [12], water buffaloes [34,35], and goats [24], but data on other bovids remain scarce. For example, in dairy cattle, 10 protein variants for α-S1 casein, 5 for α-S2 casein, 15 for β-casein, and 11 for κ-casein have been documented [36]. In addition to these protein variants, sequence variations have been identified in the upstream and downstream gene regions, which can affect casein gene expression and influence the amount and ratio of different caseins in goat milk [37,38,39,40].

A more comprehensive understanding of the genetic diversity of milk protein genes across a wider range of Bovidae species could offer valuable insights into the evolution of the casein cluster, especially in differentiating wild from domestic bovids. However, this effort has been hindered by the lack of genome-wide SNP data for most wild members of the family and the absence of species-specific SNP microarrays for comparative analysis. Currently, SNP microarrays are only available for domesticated species: domestic cattle, water buffalo [41], and sheep [42]. In this study, the challenge of the missing species-specific SNP microarrays was addressed by using the Illumina Bovine MDv2 Chip. The use of cattle-derived SNP chips proved effective across diverse Bovidae species, with over 83% of SNPs successfully genotyped (Table 2). This finding is consistent with previously reported data; for example, when genotyping water buffalo using the Illumina BovineSNP50 BeadChip, 41,870 of the 54,001 SNPs were successfully genotyped, and 1159 bovine SNPs remained polymorphic in the species [43].

To date, the Illumina BovineSNP50 BeadChip has been successfully applied to genotyping gaur (*Bos gaurus*), banteng (*B. javanicus*), yak (*B. grunniens*), African buffalo (*S. caffer*), and lowland anoa (*Bubalus depressicornis*) [44]; European bison (*Bison bonasus*) and American bison (*B. bison*) [44,45]; and the scimitar-horned oryx (*Oryx dammah*) and Arabian oryx (*O. leucoryx*) [46]. SNPs represented on the Illumina Bovine50K BeadChip are evenly distributed across each cattle chromosome. However, this may not be the case for other species. For instance, in water buffalo, over 14,000 genes lack SNP coverage on the current BeadChip [43]. Conserved cross-species SNPs may limit the chip’s effectiveness in identifying runs of homozygosity (ROH) or estimating linkage disequilibrium (LD) [47]. In our study, 10 randomly chosen Bovine BeadChip probes showed high similarity to genomic sequences of bovids in the range above 86% for at least 98% of the probes (Table 2). This further supports our decision to use this microarray to analyze genetic diversity within the family.

The highest polymorphism levels were found in the genus *Bos* and lechwe (*K. leche*) (Table 4), aligning with previous findings of high genetic diversity in cattle and zebu (*B. indicus*), showing high polymorphism at both the casein gene and whole-genome levels [48,49,50]. In *K. leche*, the unexpectedly high level of polymorphism, comparable to that of domestic cattle, may be due to the presence of 5 subspecies [51,52], thus contributing significant genetic variability. Similarly, high genetic diversity has been reported in the mitochondrial DNA of the African bushbuck (*Tragelaphus scriptus*), which also consists of several subspecies [53].

The uniqueness of lechwe may be linked to their adaptations to semi-aquatic wetland environments [54]. They have elongated hooves, a water-repellent coat, and are strong swimmers, using water bodies as a refuge from predators. This wet diet, rich in water and nutrients such as β-carotene, fatty acids, and certain vitamins, can enhance the nutritional quality of their milk by affecting fat content, protein levels, and overall energy. Such a nutrient-rich diet may influence the expression of casein genes and, consequently, the casein content in their milk [55,56]. For wetland-adapted species, there may be evolutionary adaptations that link their diet to milk biosynthesis, ensuring that offspring receive adequate nutrition in a water-rich but potentially less energy-dense environment [57,58]. Interestingly, lechwe milk contains the highest mono-unsaturated fatty acid (MUFA) content among several ruminants [28], which could be particularly beneficial for energy and nutrient absorption, as well as reducing inflammation. The recent sequencing of the red lechwe (*K. leche*) genome may significantly advance research on this unique species [59].

In our phylogenetic analyses based on SNPs located in the 5′-upstream region and the 3′-downstream region (Figure 3A,C), we identified two main clusters, with one including only members of the genus *Bos* (domestic cattle, watusi, and zebu). This result is not surprising, as *Bos* is one of the few bovid genera (along with *Bubalus*, *Capra*, *Ovis*, and *Rangifer*) that have been extensively domesticated and bred by humans for thousands of years. *Bos* species are found worldwide and are known for their high genetic diversity and specialized traits, including milk production, meat, draft power, and other uses. Similarly, water buffalo (genus *Bubalus*), although not included in our study, is another domesticated species heavily relied upon for dairy production, particularly in South Asia, where breeds like the Murrah and Nili-Ravi are known for their high milk yields [41,60,61]. In another analysis based on SNPs located in the casein gene cluster, the *Bos* cluster was weakly joined by *S. caffer* (Figure 3B). Although this relationship should be interpreted with caution, the genera *Bos* and *Syncerus* share several characteristics and stand out from many other bovid genera due to their large size, grazing habits, social structures, and adaptations to diverse habitats.

In the subfamily Hippotraginae, intraspecies sequence divergence has only been studied in the roan antelope (*Hippotragus equinus*) [62] and sable antelope (*H. niger*) [63]. In 5 re-sequenced roan antelopes, 3.4 million single-nucleotide variations (SNVs) were identified, with homozygous SNVs ranging from 577,765 to 949,845 and heterozygous SNVs from 711,962 to 1,043,928 [62]. In contrast, the sable antelope showed lower diversity, with homozygous SNVs between 260,651 and 377,251, and heterozygous SNVs from 464,813 to 597,659 [63].

Associations between polymorphisms of milk-encoding genes and milk production traits have been the focus of numerous studies in dairy cattle, e.g., [64,65,66]. The most notable effects of the milk protein polymorphisms on economically important traits are their influence on casein content and the cheesemaking properties of milk, primarily in modern breeds of dairy cattle [12]. Similar studies, including production traits such as milk yield and milk composition, were also conducted on water buffalo, e.g., [67,68,69]. Differences in gene promoters and enhancers might explain why water buffaloes produce less milk (5 to 10 times less) but higher-quality milk than cattle [43]. Venturini [70] identified 1562 SNPs associated with milk production and/or quality. Asim [67] demonstrated associations between milk yield and genetic variants in the *Csn1s1*, *Csn2*, *Csn3*, and *Blg* genes in both cattle and water buffaloes. Pauciullo et al. [69] validated and confirmed the association of three SNPs in key genes (*Csn1*, *Csn3*, and *LPL*) with milk yield, protein, and fat. Medeiros [68] reported an association between a SNP located in the promoter region of *Csn1s1* at position -258 (A/G) and identified variants that may affect the gene’s expression.

Sequence variation in transcription factor binding sites can alter the timing, location, and level of gene expression. Analysis of 5′-upstream sequences in 28 milk protein genes revealed C/EBP, CTF/NF1, MAF, and MGF (STAT5) transcription factor binding sites in all of them [71,72]. Comparing high-milk-output cattle with low-yield species like sheep and camels showed that YY1 binding sites are more frequent in sheep than in camels and very sparse in cattle, suggesting that the ratio of YY1 sites may influence milk production [73].

A core composite response element, which primarily controls milk protein gene activity, was identified through a search for elements conserved within all 5′-flanking sequences analyzed in cows and camels. This element, which contains two motifs with essential transcription factor binding sites, likely regulates gene expression in the lactating mammary gland [74]. Intragenic sequences of some major milk protein-encoding genes contribute to hormonal regulation and contain important regulatory elements. They have been identified within introns [75,76] and/or in the 3′ untranslated region (UTR) and flanking regions [77].

Although 3′ flanking regions receive less attention than 5′ promoters, they are also important for regulating casein gene expression at the protein level. In the mammary gland, post-transcriptional regulation, particularly mRNA stability, plays a role in milk protein expression. For instance, miR-101a suppresses β-casein mRNA expression during differentiation and involution in mouse mammary tissue and cells [78]. Various miRNAs (e.g., miR-15a, miR-139, miR-423-5p) affect milk protein synthesis by regulating key genes involved in these pathways [79].

Improving dairy traits has long been a key goal of breeding programs, especially in the genomic selection era. Understanding genetic variations in the casein gene cluster, particularly those related to milk biosynthesis, can help breeders select for traits that enhance milk yield, quality, and adaptability. Such advancements contribute to both food security and economic stability. Although our samples offer valuable insights into the genetic diversity of the casein gene cluster, the limited sample size may not fully reflect the broader genetic variation found in wild populations. Future research with larger and more geographically diverse samples will be crucial to validating and extending these findings, providing a more comprehensive understanding of genetic diversity in both wild and domestic Bovidae.

## 5. Conclusions

The bovine SNP microarray is a powerful tool for studying genetic diversity within the Bovidae family, highlighting variations in the casein gene cluster, particularly in the 5′ flanking region. The observed differences in genetic diversity within the casein gene cluster suggest potential variability in milk protein expression between wild and domestic species, though further studies are needed to directly link these genetic variations to functional outcomes in milk biosynthesis. The relatively low cost of SNP microarrays makes genomic analysis applicable across a wide range of research areas.

## Figures and Tables

**Table 1:** Probes selected to test their similarity to the genome sequences of Bovidae.

Probe Name	Sequence
EuroG10K_Hapmap 60224	CCAACCAGAGGTCCACCAGAGTCACCCTAGAGAGAAAAGGATATATATAA
Hapmap 59186	CCATGTGAGTGTCAATGTTGGCTTGAACAGTGTTGTTTTCCTGGCAACCA
Hapmap 25708-BTC-043671	CTAGAAGTCCTCCTGAGAAGACATAAGAGAAAAGCCAAAGACACTGTCAT
Hapmap 24184-BTC-070077	GAGCACAACAAATTATAACTGCAAAGCATCAAAAAGACCACATCAAACAC
BTA-111108-no-rs	TTAATAAACAGTAAAGGTCAGGCAGATGGTCCCTCTCCCTCACATATTCA
Hapmap 52348	TACTTTCCACCTTATTTATTACCCAGAGCCACACAGTTAAGCAAGCGATT
BTA-77173-no-rs	GCATACAGGATAAATTATAAATGTGTGCCCATTATAATAAGGCCTGCAAA
ARS-BFGL-NGS-2418	GTTTTTGTTTTTTATTTCACTTTCAGGAAGGCCCACTACAATGGCAGAGT
BTA-118117	AAATAAGATGTGATGTTTAAGAGACACTGATGGAACCTGGTGCTTATTTA
BTB-00268331	CGCCTGGCAAGAACTGCCATCCTAAAACATACCCAAAGTTGCCTTGGATT

**Table 2:** Identity (%) of the Illumina bovine probe with genomic DNA of different Bovidae species.

Species	GenBank Accession No	EuroG10KHapmap 60224	Hapmap 59186	Hapmap 25708-BTC-043671	Hapmap 24184-BTC-070077	Hapmap 25779-BTC-072896	Hapmap 52348	BTA-77173-no-rs	ARS-BFGL-NGS-2418	BTA-118117	BTB-00268331
*B. indicus*	GCF_000247795.1	100	100	100	96	98	100	100	100	100	100
*A. melampus*	GCA_006408695.1	100	96	96	94	ns	92	96 **	90	86 **	92
*C. taurinus*	GCA_006408615.1	100	96	90 **	94 **	92	93 * (80)	96	92 * (60)	90 **	92
*S. caffer*	GCA_006408785.2	98	96	96	100	98	98	100	98	100	88
*T. imberbis*	GCA_006410775.1	98	88	94	94	92	94	98	92	94	92 * (80)
*H. niger*	GCA_006942125.1	100	98	92	92	90	98	92	92	94 * (60)	92
*K. leche*	GCA_014926565.1	100	96	94	92	94 * (80)	98	98	92 * (80)	94 * (40)	90
*K. ellipsiprymnus*	GCA_006410655.1	100	96	94	92 * (80)	94	98	100	92	94 * (20)	88

*―SNP called only in a portion of samples (in parentheses, % of samples), ns―no-call in all samples, **―nucleotide non-compatible with the genomic sequence at the probe 3′ end.

**Table 3:** Similarity of the probe Hapmap 24184-BTC-070077 sequence to analyze genomic sequences. The 3′-nucleotide is underlined, and the sequence of *C. taurinus* with an incompatible last 3′-nucleotide is shaded.

Species	Probe Sequence
*B. taurus*	GAGCACAACAAATTATAACTGCAAAGCATCAAAAAGACCACATCAAACAC
*B. indicus*	........................................Y.W.......
*A. melampus*	...G............G.........................C.......
*C. taurinus*	...G............G................................T
*S. caffer*	..................................................
*T. imberbis*	...............CG..A..............................
*H. niger*	...G....A.......G...T.............................
*K. leche*	................G..........T..........TG..........
*K. ellipsiprymnus*	................G..........T..........TG..........

**Table 4:** SNP call rates (%) in 12 analyzed taxa. Note that domestic cattle and watusi belong to the same species, *B. taurus*.

Species	*n*	Call Rate	
		All SNPs	Casein Gene Cluster
*A. melampus*	5	83.0	82.9
*C. taurinus*	5	85.1	84.2
Domestic cattle	12	100.0	100.0
Watusi	5	98.3	98.0
*B. indicus* (zebu)	5	99.5	99.5
*S. caffer*	9	95.0	94.5
*T. angasii*	5	88.7	87.5
*T. imberbis*	6	89.6	87.6
*H. equinus*	5	87.1	87.1
*H. niger*	5	87.9	87.6
*K. leche*	5	83.1	84.0
*K. ellipsiprymnus*	5	83.3	83.6

**Table 5:** Percentage of polymorphic loci in the casein gene cluster and its 5′-upstream and 3′-dowstream regions in individual species and genera. Note that domestic cattle and watusi belong to the same species, *B. taurus*.

Taxon	5′-Upstream Total	Casein Gene Cluster				3′-Downstream Total
		Total	Exons	Introns	Intergenic	
Species						
*A. melampus*	13.64	8.89	8.57	0.00	28.57	8.51
*C. taurinus*	27.27	17.78	20.00	16.67	14.29	6.38
Domestic cattle	68.18	37.78	34.29	50.00	71.43	89.36
Watusi	68.18	40.00	37.14	33.33	57.14	53.19
*B. indicus* (zebu)	63.64	35.56	28.57	50.00	42.86	40.43
*S. caffer*	18.18	15.56	14.29	16.67	28.57	8.51
*T. imberbis*	9.09	11.11	14.29	0.00	14.29	8.51
*T. angasii*	18.18	20.00	22.86	16.67	0.00	10.64
*H. equinus*	22.73	15.56	17.14	0.00	28.57	6.38
*H. niger*	18.18	15.56	17.14	0.00	28.57	6.38
*K. leche*	54.55	55.56	57.14	33.33	71.43	36.17
*K. ellipsiprymnus*	9.09	13.33	14.29	0.00	28.57	4.26
Genus						
*Bos*	90.91	55.56	51.43	50.00	85.71	91.49
*Tragelaphus*	18.18	26.67	28.57	16.67	28.57	17.02
*Hippotragus*	22.72	20.00	22.86	0.00	28.57	8.51
*Kobus*	54.54	60.00	62.86	33.33	71.43	38.30
Mean	30.42	22.56	22.64	16.67	31.87	21.27
SE	6.62	4.07	3.78	5.34	6.30	7.20

**Table 6:** Summary of the genetic diversity indices (mean ± SE) of (A) 22 SNPs in the 5′-upstream region, (B–E) 45 SNPs in the casein gene cluster, and (F) 47 SNPs in the 3′-downstream region. Note that domestic cattle and watusi belong to the same species, *B. taurus*.

Taxon	Ho	He	uHe	Fst
A. 5′-upstream region				
*A. melampus*	0.121 ± 0.068	0.066 ± 0.036	0.077 ± 0.042	−0.833 ± 0.062
*C. taurinus*	0.205 ± 0.085	0.123 ± 0.044	0.186 ± 0.072	−0.556 ± 0.172
Domestic cattle	0.223 ± 0.051	0.230 ± 0.045	0.240 ± 0.047	0.028 ± 0.078
Watusi	0.373 ± 0.075	0.275 ± 0.045	0.309 ± 0.051	−0.322 ± 0.104
*B. indicus* (zebu)	0.232 ± 0.050	0.197 ± 0.039	0.220 ± 0.043	−0.164 ± 0.058
*S. caffer*	0.091 ± 0.063	0.083 ± 0.039	0.109 ± 0.055	0.000 ± 0.246
*T. angasii*	0.066 ± 0.047	0.055 ± 0.028	0.062 ± 0.031	−0.063 ± 0.175
*T. imberbis*	0.045 ± 0.045	0.037 ± 0.026	0.041 ± 0.029	0.000 ± 0.302
*H. equinus*	0.152 ± 0.075	0.105 ± 0.043	0.144 ± 0.062	−0.333 ± 0.201
*H. niger*	0.125 ± 0.065	0.072 ± 0.035	0.106 ± 0.055	−0.619 ± 0.095
*K. leche*	0.232 ± 0.077	0.196 ± 0.044	0.249 ± 0.061	−0.069 ± 0.163
*K. ellipsiprymnus*	0.091 ± 0.063	0.045 ± 0.031	0.052 ± 0.036	−1.000 ± 0.000
Total	0.163 ± 0.019	0.124 ± 0.012	0.150 ± 0.015	−0.230 ± 0.039
B. Total casein gene cluster				
*A. melampus*	0.089 ± 0.043	0.044 ± 0.021	0.074 ± 0.037	−1.000 ± 0.000
*C. taurinus*	0.172 ± 0.056	0.088 ± 0.029	0.141 ± 0.048	−0.950 ± 0.021
Domestic cattle	0.122 ± 0.029	0.112 ± 0.026	0.117 ± 0.027	−0.082 ± 0.023
Watusi	0.188 ± 0.042	0.151 ± 0.030	0.169 ± 0.034	−0.227 ± 0.065
*B. indicus* (zebu)	0.151 ± 0.036	0.124 ± 0.027	0.138 ± 0.030	−0.200 ± 0.050
*S. caffer*	0.077 ± 0.035	0.047 ± 0.019	0.057 ± 0.024	−0.429 ± 0.065
*T. angasii*	0.093 ± 0.038	0.061 ± 0.021	0.079 ± 0.030	−0.349 ± 0.077
*T. imberbis*	0.042 ± 0.024	0.033 ± 0.016	0.040 ± 0.019	−0.196 ± 0.075
*H. equinus*	0.104 ± 0.044	0.064 ± 0.023	0.094 ± 0.037	−0.492 ± 0.113
*H. niger*	0.144 ± 0.052	0.075 ± 0.026	0.122 ± 0.044	−0.905 ± 0.038
*K. leche*	0.150 ± 0.040	0.175 ± 0.026	0.207 ± 0.032	0.196 ± 0.106
*K. ellipsiprymnus*	0.122 ± 0.048	0.064 ± 0.025	0.104 ± 0.042	−0.889 ± 0.041
Total	0.118 ± 0.011	0.083 ± 0.007	0.110 ± 0.010	−0.307 ± 0.024
C. Exon casein gene cluster				
*A. melampus*	0.086 ± 0.048	0.043 ± 0.024	0.067 ± 0.038	−1.000 ± 0.000
*C. taurinus*	0.193 ± 0.066	0.099 ± 0.034	0.153 ± 0.055	−0.943 ± 0.026
Domestic cattle	0.088 ± 0.026	0.089 ± 0.027	0.093 ± 0.028	−0.013 ± 0.015
Watusi	0.171 ± 0.047	0.138 ± 0.033	0.154 ± 0.037	−0.225 ± 0.076
*B. indicus* (zebu)	0.109 ± 0.033	0.093 ± 0.027	0.104 ± 0.030	−0.160 ± 0.045
*S. caffer*	0.085 ± 0.044	0.048 ± 0.024	0.059 ± 0.030	−0.545 ± 0.079
*T. angasii*	0.114 ± 0.048	0.073 ± 0.026	0.095 ± 0.037	−0.379 ± 0.092
*T. imberbis*	0.054 ± 0.030	0.042 ± 0.020	0.051 ± 0.024	−0.196 ± 0.085
*H. equinus*	0.106 ± 0.049	0.068 ± 0.027	0.092 ± 0.039	−0.407 ± 0.134
*H. niger*	0.157 ± 0.061	0.082 ± 0.031	0.138 ± 0.054	−0.889 ± 0.046
*K. leche*	0.155 ± 0.048	0.187 ± 0.030	0.222 ± 0.037	0.239 ± 0.126
*K. ellipsiprymnus*	0.129 ± 0.056	0.068 ± 0.029	0.106 ± 0.047	−0.867 ± 0.050
Total	0.121 ± 0.014	0.086 ± 0.008	0.111 ± 0.011	−0.284 ± 0.030
D. Intron casein gene cluster				
*A. melampus*	0.000	0.000	0.000	
*C. taurinus*	0.167 ± 0.167	0.083 ± 0.083	0.167 ± 0.167	−1.000 ± 0.167
Domestic cattle	0.250 ± 0.120	0.190 ± 0.089	0.198 ± 0.093	−0.302 ± 0.037
Watusi	0.200 ± 0.126	0.140 ± 0.089	0.156 ± 0.098	−0.429 ± 0.000
*B. indicus* (zebu)	0.333 ± 0.161	0.213 ± 0.098	0.237 ± 0.109	−0.528 ± 0.098
*S. caffer*	0.056 ± 0.056	0.046 ± 0.046	0.056 ± 0.056	−0.200 ± 0.056
*T. angasii*	0.033 ± 0.033	0.030 ± 0.030	0.033 ± 0.033	−0.111 ± 0.033
*T. imberbis*	0.000	0.000	0.000	
*H. equinus*	0.000	0.000	0.000	
*H. niger*	0.000	0.000	0.000	
*K. leche*	0.042 ± 0.042	0.090 ± 0.058	0.101 ± 0.065	0.429 ± 0.330
*K. ellipsiprymnus*	0.000	0.000	0.000	
Total	0.090 ± 0.027	0.066 ± 0.018	0.079 ± 0.022	−0.292 ± 0.055
E. Intergenic casein gene cluster				
*A. melampus*	0.286 ± 0.184	0.143 ± 0.092	0.238 ± 0.158	−1.000 ± 0.000
*C. taurinus*	0.143 ± 0.143	0.071 ± 0.071	0.143 ± 0.143	−1.000 ± 0.143
Domestic cattle	0.226 ± 0.077	0.219 ± 0.074	0.228 ± 0.077	−0.056 ± 0.080
Watusi	0.207 ± 0.088	0.198 ± 0.078	0.223 ± 0.088	−0.081 ± 0.146
*B. indicus* (zebu)	0.086 ± 0.040	0.111 ± 0.061	0.124 ± 0.067	0.101 ± 0.139
*S. caffer*	0.286 ± 0.184	0.143 ± 0.092	0.190 ± 0.123	−1.000 ± 0.000
*T. angasii*	0.000	0.000	0.000	
*T. imberbis*	0.036 ± 0.036	0.031 ± 0.031	0.036 ± 0.036	−0.143 ± 0.036
*H. equinus*	0.286 ± 0.184	0.143 ± 0.092	0.229 ± 0.154	−1.000 ± 0.000
*H. niger*	0.286 ± 0.184	0.143 ± 0.092	0.190 ± 0.123	−1.000 ± 0.000
*K. leche*	0.195 ± 0.106	0.234 ± 0.068	0.278 ± 0.083	0.211 ± 0.277
*K. ellipsiprymnus*	0.214 ± 0.149	0.125 ± 0.082	0.214 ± 0.149	−0.667 ± 0.178
Total	0.167 ± 0.034	0.121 ± 0.020	0.162 ± 0.030	−0.271 ± 0.063
F. 3′-downstream region				
*A. melampus*	0.080 ± 0.039	0.040 ± 0.019	0.072 ± 0.036	−1.000 ± 0.000
*C. taurinus*	0.056 ± 0.032	0.030 ± 0.017	0.051 ± 0.030	−0.889 ± 0.027
Domestic cattle	0.350 ± 0.031	0.313 ± 0.024	0.326 ± 0.025	−0.101 ± 0.033
Watusi	0.235 ± 0.038	0.205 ± 0.029	0.230 ± 0.032	−0.160 ± 0.061
*B. indicus* (zebu)	0.143 ± 0.032	0.149 ± 0.027	0.166 ± 0.030	0.052 ± 0.068
*S. caffer*	0.067 ± 0.035	0.037 ± 0.018	0.050 ± 0.027	−0.723 ± 0.057
*T. angasii*	0.084 ± 0.039	0.044 ± 0.020	0.067 ± 0.032	−0.822 ± 0.056
*T. imberbis*	0.080 ± 0.039	0.040 ± 0.019	0.071 ± 0.035	−1.000 ± 0.000
*H. equinus*	0.041 ± 0.025	0.025 ± 0.015	0.038 ± 0.023	−0.583 ± 0.054
*H. niger*	0.060 ± 0.034	0.030 ± 0.017	0.052 ± 0.030	−1.000 ± 0.000
*K. leche*	0.074 ± 0.030	0.103 ± 0.022	0.132 ± 0.033	0.314 ± 0.102
*K. ellipsiprymnus*	0.040 ± 0.028	0.020 ± 0.014	0.032 ± 0.023	−1.000 ± 0.000
Total	0.110 ± 0.011	0.086 ± 0.007	0.108 ± 0.010	−0.202 ± 0.023

Abbreviations: Ho―heterozygosity observed, He―heterozygosity expected, uHe―unbiased heterozygosity expected, Fst―fixation index.

## Data Availability

The original contributions presented in this study are included in the article and Supplementary Materials. Further inquiries can be directed to the corresponding author. The raw data supporting the conclusions of this article are available from the first author upon reasonable request.

## Supplementary Materials

The following supporting information can be downloaded at: https://www.mdpi.com/article/10.3390/ani14203034/s1, Table S1: Genotypes and annotation of SNPs localized in the casein gene cluster and 2 Mb of upstream and downstream regions; Table S2: Nei’s genetic distances (below the diagonal) and pairwise F-estimates (above the diagonal) among 12 analyzed Bovidae taxa.
