# Supplementary material for: Molecular Diversity of the Casein Gene Cluster in Bovidae: Insights from SNP Microarray Analysis
Source: Animals (Basel). 2024 Oct 19;14(20):3034. doi: 10.3390/ani14203034 (PMC11505306; doi:10.3390/ani14203034)
Supplement: Supplementary file 1 [file animals-14-03034-s001.zip › Animals_Malewski et al_Table S2_17-10-2024.pdf]

Table S2. Nei's genetic distance (below diagonal) and pairwise *F*-estimates (above diagonal). Note that domestic cattle and watusi belong to one species — *B. taurus*

Taxon	*H. equinus*	*H. niger*	*S. caffer*	Domestic cattle	*C. taurinus*	*A. melampus*	*K. leche*	*K. ellipsiprymnus*	*T. imberbis*	*T. angasii*	Watusi	*B. indicus*
H. equinus		0.325	0.599	0.447	0.446	0.647	0.205	0.545	0.621	0.623	0.325	0.355
H. niger	0.097		0.527	0.447	0.496	0.634	0.219	0.549	0.559	0.774	0.312	0.343
S. caffer	0.364	0.326		0.461	0.643	0.656	0.389	0.644	0.631	0.641	0.421	0.358
Domestic cattle	0.541	0.521	0.574		0.481	0.538	0.317	0.516	0.487	0.473	0.204	0.173
C. taurinus	0.187	0.223	0.494	0.618		0.659	0.314	0.614	0.665	0.664	0.393	0.413
A. melampus	0.365	0.342	0.362	0.726	0.411		0.379	0.772	0.771	0.762	0.475	0.394
K. leche	0.079	0.084	0.345	0.353	0.201	0.312		0.326	0.375	0.368	0.229	0.253
K. ellipsiprymnus	0.216	0.183	0.347	0.616	0.319	0.362	0.201		0.758	0.751	0.397	0.432
T. imberbis	0.294	0.246	0.260	0.586	0.447	0.398	0.270	0.383		0.611	0.432	0.323
T. angasii	0.346	0.298	0.315	0.580	0.511	0.454	0.288	0.437	0.256		0.368	0.386
Watusi	0.287	0.250	0.412	0.201	0.473	0.569	0.186	0.382	0.373	0.297		0.195
B. indicus	0.354	0.320	0.318	0.150	0.512	0.422	0.229	0.441	0.261	0.348	0.137	
